# Hospital and Institutional News

**Published:** 1916-12-02

**Authors:** 


### HOSPITAL AND INSTITUTIONAL NEWS.

### DEATH OF SIR GEORGE WHITE.

By the death of Sir George White the Bristol Royal Infirmary loses a President and Treasurer whom it will be very difficult to replace; while the Bristol Red Cross organisation will also sorely miss his organising genius and hard-working devotion to the cause of the wounded. Sir George White, who was born in 1854, was a stockbroker originally; but success in this profession was not enough to occupy all his energies, and he did a great deal, in conjunction with Sir J. Clifton Robinson, to extend the construction of electric tramways in various parts of the kingdom. When flight came within the range of practical business enterprise Sir George White was one of the first to see its commercial possibilities; and the well-known Bristol aircraft works were very largely his creation. In spite of all these preoccupations, Sir George White found time to devote a great deal of valued service to the Bristol Royal Infirmary, to the presidency and treasurership of which he was elected in 1904; and he was also President of the Queen Victoria Memorial Hospital at Nice. Among the philanthropists of provincial cities who devoted their time and resources to the assistance of the voluntary system Sir George White will always take a high place. Bristol, in particular, owes him a debt of gratitude which should ensure his memory remaining green there for very many years. He was created a baronet in 1904, and the title descends to his only son, who was born in 1882. A full account of Sir George White's work for the Royal Infirmary appeared in our columns on May 20, 1911, page 189, in the Eminent Chairmen series.

### THE SINKING OF HOSPITAL SHIPS.

If the sinking of the *Britannic* and of the *Braemar Castle* was the work, as is apparently the case, of German or Austrian submarines, one more of the provisions of the Geneva Convention has been thrown over by the ruthless enemies of civilisation. It is true that the Germans have not yet reached the stage of declaring open and unconcealed war on hospital ships, though they or their allies have already sunk two Russian Red Cross boats and have attempted to sink the *Asturias*. But for many months now their Government has repeatedly published hints and innuendos to the effect that the Allies are using hospital ships for improper purposes; and these baseless fabrications can only have been concocted as excuses for future outrages of the type committed during the past week. It looks as if we have to reckon with a regular campaign of destruction against hospital ships; and as if the distinguishing marks and lights of these vessels may merely serve the purpose of helping the assassins to select their victims. Meanwhile it is noteworthy that all accounts of the sinking of the *Britannic* concur in praising the cool heroism of the nursing staff on board her, as well as of the doctors and orderlies of the Royal Army Medical Corps.

### BROWNLOW HILL INFIRMARY.

In The Hospital of March 18, 1916, we published a paragraph in Institutional News relating to a report of Dr. Evans, visiting surgeon of Brownlow Hill Infirmary, Liverpool. At that time the writer of the paragraph misapprehended the bearing of Dr. Evans' report, for which, in the absence of a copy, we were dependent upon newspaper reports. The writer of the paragraph was misled into the belief that the reference to the increase of venereal disease in Dr. Evans' report related to the whole of the female division of the infirmary, and not to the female Lock wards only. We find from the full copy of Dr. Evans' report that his observations referred to the female Lock wards in particular, and not to the maternity wards or to the whole of the female division of the infirmary, and we gladly withdraw the inferences based on Dr. Evans' supposed report. Dr. Evans in his report made no reflection upon the maternity wards, and we have to express our regret that any reflection on the Select Vestry should have appeared in this connection in The Hospital of March 18, and to apologise to the members of that body for the observations published consequent upon the error into which the writer fell in the circumstances mentioned.

### THE LATE DR. DOYEN.

The death of Eugène Louis Doyen at the age of fifty-seven removes from the world of surgery one of its most forceful and picturesque, if not one of its most judicious or single-minded, exponents. In early days Doyen is said to have refused to comply with the conditions laid down by Pasteur before the latter would accept the young man as his assistant; and if the story is true it shows that Doyen was as self-confident and independent then as afterwards. Self-confidence, carried even to overweening lengths, seems to have been the keynote of his career. Throughout his professional life Doyen cared little for the esteem of his French colleagues, and nothing at all for their opinion of his theories. As an operator he was boldness itself, and by his personality and his *penchant* for the limelight of popular attention he succeeded in building up an enormous private practice. He maintained that research was best carried out by one in the position of a busy practical worker; if his researches had been of the value that he attributed to them, this theorem might have gained more ground than it has done. Fertile in imagination and insufficiently inductive in method, Doyen committed himself to several sensational dogmata which time and his colleagues abundantly disproved. He discovered, as he thought, the parasite of cancer; and prepared from it a serum which he said was curative of that disease. Both the parasite and the value of the serum turned out to be fallacious, though not before Doyen had reaped enormous profits from their exploitation: it is said that he took a fee of £8,000 from a wealthy American for treating his wife, who died not long after in spite of Doyen's assurances that he could cure her. Less than four years ago this stormy petrel of surgery invented an "elixir of life" which was curative of nearly a hundred diseases. In spite of genuine contributions to the advancement of surgery and of many great gifts, Doyen is probably fated to remain in memory chiefly as a charlatan of an unusual and somewhat original type.

### THE LATE MR. CHARLES BOOTH.

The death is announced of the well-known social reformer, Mr. Charles Booth, at the age of seventy-six. Until he was forty-six years old Mr. Booth devoted the whole of his time to the affairs of the family shipping business of Alfred Booth & Co. Later, and till the time of his death, he was on the board of the Booth Steamship Company, of which he became chairman. In 1886 he began his great work of investigating the social conditions of the life of the poor, and in 1889 he published a volume entitled "Life and Labour of the People in East London," which had a tremendous influence in arousing public attention and in interesting the well-to-do classes in the welfare of their poorer neighbours. From this time onwards Mr. Booth was a recognised authority on social reform, and for many years he continued his publication of books bearing upon this topic. He is described as one who "said what he believed, not what he wished others to believe. He searched diligently for the truth, the whole truth, and nothing but the truth, and the world is the poorer for his death." Mr. Booth was made a Privy Councillor in 1904, and received honorary degrees from the Universities of Oxford, Cambridge, and Liverpool. He was also a member of Mr. Chamberlain's Tariff Commission in 1904.

### A SWORD FOR A FUND'S VICE-CHAIRMAN.

Mr. Sam Ingham, vice-chairman of the Bradford Hospital and Convalescent Fund, has been presented with a sword by the officers of the Fund and his friends on having become a platoon commander of the 5th Battalion West Riding Volunteers. During the past fourteen years Mr. Ingham has occupied in turn almost every responsible post connected with the Fund, and the presentation expresses the affection and gratitude of his colleagues.

### ORTHOPÆDIC SURGERY FOR THE WOUNDED.

King Manoel of Portugal, who is evincing a deep interest in the wounded of the Allies, and particularly in limbless men in special hospitals in this country, is continuing his round of visits to important centres by proceeding to Leeds shortly. In that city he will be the guest of Sir Berkeley Moynihan, and will inspect the orthopædic work in progress there. Later, King Manoel will go to Liverpool and, accompanied by Sir Walter Lawrence and Colonel Robert Jones, will make a similar tour of inspection. Scotland was lately visited on a similar mission, with results which are already well in train. The Scottish Red Cross Society is proceeding with a scheme in connection with the Bellahouston Red Cross Hospital at Glasgow, at an estimated cost of £5,000. At Aberdeen it is believed that suitable huts and equipment at the Oldmill 1st Scottish Military Hospital will require a sum of £2,500. The Aberdeen area extends from Stonehaven to Inverness and Caithness, and it is hoped that a scheme will be established for obtaining subscriptions to meet the sum required.

### A WITTENBERG MEMORIAL.

In the summer of this year an appeal was made to the public for small subscriptions for a memorial of the three officers of the Royal Army Medical Corps who lost their lives in the horrible Wittenberg Camp in Germany—namely, Major W. B. Fry, Captain A. A. Sutcliff, and Captain Stephen Field. Lady Parker (of Waddington) has now notified the subscribers that the sums received enable her to build and furnish one of the single rooms at the Star and Garter Hospital at Richmond. She holds, and without doubt rightly, that this provision for disabled soldiers will commend itself to the subscribers as a suitable way of commemorating the heroism of Major Fry and his companions; and she further proposes to expend the balance of her fund in the erection of a tablet (on the wall of the room), recording the names of the officers and the manner of their death. If possible, we should like to see added to the roll the names also of the orderlies, no less heroic than their officers, who died in the same camp and in the same circumstances. This lasting memorial to the shame of German brutality, no less than to the British martyrs, may serve to remind future generations what they owe to their ancestors, and what German pretensions to dominate the world would have meant had they been allowed to materialise.

### AN X-RAY VICTIM AT MIDDLESEX HOSPITAL.

With the discovery and universal adoption of methods of protection for *x*-ray workers against the risks of dermatitis and of malignant diseases, the mortality amongst the early pioneers of radiology has been practically abolished. It is seldom, indeed, that additions to the death-roll have to be chronicled nowadays; for the early workers who suffered burns are, for the most part, long since recovered, or, in the minority of cases, already dead. On November 22 Mr. R. F. Mann was buried at Harrow, after five operations for *x*-ray dermatitis and seventeen years of work in the *x*-ray department of the Middlesex Hospital. Although he had been advised to relinquish his duties, Mr. Mann persisted until last July, and even did a lot of extra work in radiographing wounded soldiers. He was only thirty-five years of age, and had thus passed half his short lifetime in the *x*-ray rooms of the Middlesex Hospital.

### A NEW TEST FOR ENTERIC FEVERS.

A new test, both simple of application and highly discriminatory, is described in the *British Medical Journal* by Captain H. F. Marris, R.A.M.C., whereby the diagnosis of the enteric fevers is very greatly facilitated. It remains to be seen whether this observer's conclusions are confirmed by the experience of others; if it is, Captain Marris will certainly have lightened the burden of the clinician in war-time in a very marked degree. Briefly, the test consists in the subcutaneous injection of one thirty-third of a grain of atropine. In normal persons and in those suffering from any disease except one of the enteric fevers, this proceeding (carried out with the patient at rest) results about twenty-five minutes later in a marked acceleration of the heart's rate of beat. This increased frequency of the pulse rate lasts for about an hour, passing off gradually; the maximum increase is anything from twenty to forty beats per minute. In the enteric fevers this considerable increase is not seen; a moderate increase up to twenty beats, but usually less, may occur. Captain Marris has failed to find the atropine reaction (or rather want of reaction) in a variety of other diseases, and believes there is something specific at the bottom of the phenomenon. In carriers not actually suffering from enteric disease in an active state the reaction is negative: that is, they react like normal individuals.

### SIR HIRAM MAXIM AND A HOSPITAL SECRETARY.

The late Sir Hiram Maxim at one time took an active interest in aeronautics, and about twenty years ago invented a "heavier-than-air" machine which, although it never performed any feat which could properly be described as flying, caused a large amount of curiosity and interest. There was much secrecy surrounding the invention, and all approaches to the ground where it took its trial trip were carefully safeguarded. A well-known hospital secretary had the bright idea that perhaps Sir Hiram would consent to allow visitors, on payment of a fee to be given to the hospital, and called upon the inventor at his offices in Victoria Street. Sir Hiram was a tremendously busy man, and there were a number of people in the antechamber. The great man suddenly appeared, and said: "I am sorry I can't see all of you gentlemen to-day, but if you will each kindly tell me your business in two words I will deal with what appears to me to be the most urgent." He then passed round the room. "Boiler-plates," said one; "About that lease, Sir Hiram," another; and so on. At last he reached the hospital secretary, who gravely remarked "Flying machines," and, to the envy of the crowd, secured first audience.

### A BENEFACTOR OF THE MOUNT VERNON HOSPITAL.

Since the death last week of Mr. Charles Dunell Rudd, it has been made public that he was the anonymous donor of the fine new buildings of the Mount Vernon Hospital for Consumption and Diseases of the Chest at Northwood, which look out from the hill-top there over Hertfordshire, Middlesex, and Surrey. Mr. Rudd bore the entire cost of this building, including its equipment with everything that modern science could dictate as necessary, and it is stated that his gifts to this hospital, with which he first became associated seventeen years ago, have amounted to more than £200,000. Mr. Rudd was a man of very great force of character, and had an interesting career. In the early 'seventies he and Mr. Rhodes formed a partnership at the Kimberley Diamond Fields which resulted in the formation of the De Beers Company. It is said that Rhodes was in favour of capitalising the company at fifty thousand pounds, but that Rudd held out for one hundred thousand; the matter was settled by tossing a coin, and Rudd won. At all events, both the partners became fabulously wealthy whilst still quite young men, and both of them saw the further opportunities for million gathering which were provided by the discovery of the gold reefs at Johannesburg in 1885. Rhodes, as all the world knows, turned his thoughts and his wealth towards Empire building and power; Rudd, though a good enough Imperialist, preferred the life of a country gentleman to politics. He was twice married, and leaves a large family; indeed, it has been understood that it was the illness of one of his first wife's children that directed his interest and sympathy towards hospitals for tuberculosis in general, and the "Mount Vernon" in particular.

### BEDS TO BE DOUBLED AT NOTTINGHAM?

We have previously indicated how the present increased number of hospital beds will probably continue after the war, when an attempt should be made to reduce the size of the waiting lists and to increase the amount of hospital accommodation for civilians. A practical proof of the tendency indicated has been given at Nottingham by Mr. F. Acton, who lately urged the need for considerably extending the Nottingham General Hospital. At the date of his speech the waiting list, he said, showed no fewer than 1,590 persons seeking admission. This meant that after the war the hospital must be extended. The present accommodation for civilians must at the least be doubled. The county and city made nearly equal demands, and subscribers from both would have to combine to meet the need to which each section contributed. Once more the war has brought home to us the urgency of an insufficiency in the voluntary hospital system to which we had grown accustomed. This has created waiting lists far too big for efficiency. The truth is that after the war the beds available for civil patients may have to be doubled.

### PAYING PATIENTS IN DUNDEE.

The institution known as the Dundee Women's Hospital and Nursing Home since its removal to the present building presents a statistical report and balance-sheet of interest to all concerned in the growth of the paying-patient system in this country. The number of patients received during the past year was 165 (an increase of 19), whose payments ranged from ten shillings to three guineas a week. The daily average number of patients was 15. The income was made up of £166 from subscriptions and £836 from patients' payments, an increase, by the way, of £272 under the latter head. The total expenditure has dropped by £173 to £11,000, leaving a balance in hand of £44, which virtually extinguishes the present deficit. These accounts and statistics speak for themselves, and suggest the success of such work and the support of the patients thus provided for.

### THE EXTENSION OF HARROGATE INFIRMARY.

The recent opening of the new nurses' home, a building next door to Harrogate Infirmary, is only the first step in a more comprehensive scheme of extension. When the lease falls in, the property known as Stray Lea, in Victoria Avenue, will be handed over to the infirmary committee of management, which has taken advantage of the offer of the owner, Miss Sykes, to acquire it at a reasonable figure. At present the use which will be made of it is matter for consideration, but the site when available should prove sufficient in extent, and convenient enough in itself to meet the needs of Harrogate Infirmary for some time.

### ELLEN TERRY AT THE ROYAL EYE HOSPITAL, SOUTHWARK.

In unveiling a tablet to the memory of the late Professor Malcolm McHardy at the Royal Eye Hospital, Southwark, Miss Ellen Terry said that, as a result of his forty years' association with the hospital, Dr. McHardy had always regarded the institution as his child. She herself, who had known him long both personally and professionally, could well recall his friendship with Henry Irving, and how twenty-one years ago Irving gave a performance at the Lyceum in aid of the hospital. An interesting tribute was paid by other speakers, among whom was Mr. Keith D. Young. In his double capacity as honorary architect to the Royal Eye Hospital and as the actual designer of the memorial, Mr. Young was peculiarly fitted to have his say on the occasion.

### A DOCTOR'S EFFECTIVE INVENTION FOR SOLDIERS.

In a recent speech before the Halifax Sunday Lecture Society, Dr. C. W. Saleeby described how the new British steel helmet, adopted by the Ministry of Munitions, and largely his invention, had been the result not only of his critical suggestions concerning the French helmet, but especially of information given to him by a member of the Society's committee. This when embodied in an article was withheld by the Censor, who sent it to the Ministry of Munitions. They acted on the suggestions made, and he now possessed a sample trench helmet, which the Ministry of Munitions had given him as a memento. This was shown at the lecture and described as made of manganese steel, which is shrapnel-proof. Made in one piece, with a sloping front so as to deflect bullets, the helmet also protects against shock by an inside cap, which is surrounded by hollow rubber studs. Between every pair of these is a ventilation space, by means of which the vent in the crown of the French helmet is advantageously avoided. Dr. Saleeby added that the helmet is about to be provided with an attachment which was expected to obviate nine out of ten cases of blindness. More than that he could not say. He stated, too, that the "chin strap" was going to be worn at the back of the neck, instead of under the jaw as heretofore. The sight-preserving attachment had been invented, he said, by a medical officer; and, further, the military authorities had consented to a form of heart armour being devised. Thus we see that preventive medicine has now come to include the invention of body armour; and the medical profession may congratulate itself on the success and official recognition which its devices have already won.

### ENORMOUS CONTINGENT BEQUESTS FOR LONDON HOSPITALS AND CHARITIES.

The late Mr. Albert Gearing, who died on September 30 at the age of eighty-six, leaving an estate of the net value of £485,879, has bequeathed legacies of one hundred pounds each to thirty-one charities, chiefly hospitals. There is, however, in addition to these specific gifts, a clause in the will which may possibly result in enormous sums being ultimately divisible between no fewer than one hundred and forty hospitals and charities in London. The testator left in trust for each of his three daughters, their husbands and children, if any, sums of £40,000. In the event of failure of issue the portions are to be divided (on determination of the trust created for each) between the large number of institutions already mentioned. It has not been stated whether any or all of these three trusts are likely thus to be devoted ultimately to charitable purposes; but in the event of all three of Mr. Gearing's daughters dying childless, no less than £120,000 would become distributable to the fortunate London charities in question.

### CAMP SANITATION FOR MEDICAL RECRUITS.

In a recent public lecture in Dublin, reviewing his experiences, Captain T. Gillman Moorhead attributed some of the illness on the Eastern fronts to want of properly devised sanitation. He pointed out, and very justly, that the sudden inrush periodically made by medical men in answer to the urgent official demand for R.A.M.C. recruits led to newly qualified doctors being hastily drafted to the fronts with no previous experience in looking after a unit in the field. The result was disease, occasioned by the doctor's ignorance of camp sanitation. What was wanted, and is probably wanted still, is practical instruction in this subject for the new men in the R.A.M.C. He therefore urged the establishment of a central depôt where Regular officers of the R.A.M.C. should give practical information on all the hygienic and preventive details desirable or necessary in camp life. So many medical recruits have had to kick their heels for a wearisome period after joining that there has certainly been more time for such a course of instruction than Professor Moorhead has allowed himself to admit. And the modest week of instruction for which he asks is not enough to impress a man with the importance or scope of the latest methods of camp sanitation. Here, again, we are paying the penalty for a medical curriculum which still regards preventive medicine as, in effect, a post-graduate study. May the war modify this ancient prejudice and preventive medicine receive something more than lip homage in our medical schools!

### THE DISPENSARY CHAIN IN LONDON.

THE governors of St. Bartholomew's Hospital have made an agreement with the City of London Corporation under which a tuberculosis dispensary is being established in the City at an annual cost of not more than £700. The provision and linking-up of these dispensaries proceeds quietly, but on the whole steadily, and the time should not be distant when the chain is complete and the work co-ordinated over the London area as a whole.

### STRANGE ATTEMPTED SUICIDE.

A STRANGE case of attempted suicide with tuberculin is reported in the *Centralblatt für die gesamte Tuberkulose-Forschung* for May of this year. A female patient had been given 2 ccm. of a tuberculin preparation for self-inunction, which she secretly administered to herself by subcutaneous injection. Next day there was high fever, with quickened pulse and respiration, which only subsided after some weeks. In the lungs, however—she was a consumptive—there was no clear focal reaction, and the matter was only cleared up by the patient's confession. That such a colossal dose should not, in a known consumptive, have produced focal reaction clearly tells against the diagnostic value of tuberculin. On the other hand, as the author points out, the fact that the woman suffered no permanent aggravation of her condition is an argument against those who decry tuberculin because of its dangers. For, whether *post* or *propter*, this patient's last state was better than her first one.

### A LANCASHIRE SUPERINTENDENT ON THE MENTALLY DEFECTIVE CHILD.

SOME account of the methods employed in the training of the mentally defective with so much success at the Royal Albert Institution, Lancaster, was given a week or two ago to a Manchester Conference by Dr. W. H. Coupland, the medical superintendent. Dr. Coupland, out of all his experience, is a firm believer in complete institutional treatment as the only way of real restoration. What a defective essentially needs, he says, is removal from well-meaning but uneven and injudicious home influences. When the child goes home daily the supervision over gluttony, the inculcation of obedience and cleanliness and moral restraint are bound to be in abeyance unless the parents and the home are exceptional. Dr. Coupland has, for the same reasons, very little belief in guardianship, which at the best is but a makeshift. Anyone who has seen many relatives of mentally defective children must, he adds, have been struck by their unsuitability to give that even routine and atmosphere which are essential if progress is to be secured. Dealing with what has been accomplished at the Royal Albert Institution, Dr. Coupland says the foundation of their work is that every worker must be naturally fond of children, because all defectives, no matter what their age may be, are essentially children; that the workers must attune their minds to the outlook of the children on the world and the people around them; and that all the training must be on physiological lines. Moral association, sociability, family affinity, have to be created in the idiot; his sense of affection, like his physical sense, stands in need of development. Dr. Coupland says it should always be remembered that imbeciles had better know one thing well than merely remember something of a hundred. His stages must be short, and occupations where the end soon crowns all, and where, if possible, the purchasing by a stray visitor of his handiwork increases his interest, must be the prime factors in the curriculum. It is well to have an annual sale, therefore, and to see that patients do not fail to remind their parents of their birthdays.

### SPURRING THE PUBLIC CONSCIENCE AT PLYMOUTH.

THE two outstanding features of the Plymouth Medical Officer of Health's report are his insistence upon the urgent need for a public abattoir and a searching analysis of the infantile mortality of the borough in its relation to housing conditions. Dr. Hall points to the large amount of meat condemned and destroyed locally (over 100 tons per annum), and, in order that there may be better facilities for a more thorough inspection, expresses his earnest hope that the Corporation will seriously consider the abattoir question at an early date. With regard to infant deaths, Dr. Hall endeavours to impress upon the authority that there is a very close relation between child mortality and housing conditions and home surroundings. Over a quarter of the total deaths in the borough were those of infants under twelve months old, and in one congested district the mortality among babies reached 160.3 per 1,000. "Force of circumstances," adds the M.O.H., "compels many people to live in conditions which, while fulfilling the legal requirements, are totally incompatible with health or comfort."

### THE DEVONSHIRE HOSPITAL, BUXTON.

THE committee of the Devonshire Hospital at Buxton, led by the chairman, the Duke of Devonshire, are making a special effort to reduce the heavy debt which burdens them. Since the war began, 3,000 soldiers crippled and bent by rheumatism have received special treatment there. Two hundred are constantly under treatment. Including civilians, 4,000 patients were treated last year. The new thermal baths have been in use about a year, and have proved a great boon to large numbers of patients. There is now being installed a whirlpool bath, a system of massage in water which it is hoped will prove of great service in loosening the stiffened limbs of soldiers invalided home because of rheumatism and after gun-shot wounds. The cost of providing this installation is £11,000, and the Duke of Devonshire and his committee are appealing for this amount.

### THE CARE OF DISCHARGED SOLDIERS AT BIRMINGHAM.

An ambitious scheme for the care and training of discharged soldiers has been approved by the Birmingham Statutory War Pensions Committee. Elaborate arrangements are made under it for medical treatment. So far as domiciliary treatment is concerned, the committee professes to rely very largely upon the machinery of the National Health Insurance Act, especially as it is understood that a provision is to be effected whereby uninsured sailors and soldiers will be given equal rights with an insured person to free domiciliary medical treatment, medicines, and appliances. In certain cases ordinary medical benefit will need to be supplemented by special treatment, and it is therefore proposed to make arrangements for institutional treatment with the following, among other, agencies: General Hospital, Queen's Hospital, Royal Orthopædic and Spinal Hospital, Birmingham and Midland Eye Hospital, Birmingham Dental Hospital, Birmingham and Midland Eye and Throat Hospital, and Birmingham and Midland Skin and Lock Hospital. With regard to cases of amputation, the War Office has arranged that discharged men may attend for further treatment as out-patients at the 1st Southern General Hospital or at Dudley Road Military Hospital. Totally blind men are to be sent to St. Dunstan's Home, cases of partial blindness to the local eye hospital. Deaf men are to be taught lip-reading or some other means of communication—probably at the institution at Edgbaston. The care of tuberculous cases devolves, of course, upon the City Council, with whom it is hoped to co-operate in dealing with venereal disease. The question of the treatment of mental and epileptic cases has not yet been settled. A Birmingham home, where sailors and soldiers suffering from incurable disease or injury can be treated and, if necessary, maintained, is also suggested. There are comprehensive proposals, likewise, covering the vocational training of such men as it may be possible to restore to usefulness. The work of the Statutory War Pensions Committee has become so considerable as to necessitate the employment of a male organising secretary, at a salary of £400 per annum.

### A SOLDIER'S GIFT TO SOLDIERS.

A very nice little camp, containing twelve beds, with cheerful sun parlour and comfortably furnished, has just been opened at St. John's, Newfoundland, as a hospital for soldiers discharged from the Army suffering from tuberculosis. It has been built through the instrumentality of Mrs. Browning and a committee of ladies with money helped by the proceeds of lectures given by Private Philip Jensen, a returned soldier himself, discharged as unfit for further service, who was badly wounded in France, where the Newfoundland Regiment made itself famous. Unfit for service in the technical sense of the phrase, he is still doing good service for his King and country, devoting to them his gradually returning health and strength. He has lectured in St. John's more than once to large audiences, and is touring the island earning money for Red Cross work and gaining numbers of recruits to take the place of those who have fallen. At the close of one of his lectures a young girl came to him and asked him to accept from her a ring in aid of his fund, as she had no money and wished to give something she valued. The ring is to be raffled, and is likely to bring in a large amount of money, much more than the donor ever dreamt of.

### A TELEGRAPHIC SIGNAL SYSTEM.

A signal system of a kind used in few, if any, other similar institutions in the country has lately been installed by the Cincinnati General Hospital. It consists of a complete telegraphic outfit, the exchange, or master key, of which is placed in the main office, and more than 200 instruments are distributed through the buildings. The same wires are used for the telegraph as for the intercommunicating telephones with which the hospital is equipped, and the exchange is operated by the same person who attends to the telephone exchange. A simple number code is used and a number is given to each building and department and to each official and intern. By a manipulation of these numbers individuals may be called and dispatched to the various parts of the institution. No experience in the sending or receiving of telegraphic messages is required to operate the instruments, as no attempt is made at code words, all necessary communications having been worked out in numbers which are easily understood. The telegraphic signals are especially advantageous when the telephone fails to work or is in use, but the chief value of the system, according to the hospital officials, lies in the fact that it can be used for business only, and unnecessary conversation need not delay business messages.

### THIS WEEK'S DRUG MARKET.

The tendency of the prices of a number of drugs is still in an upward direction, although, on the other hand, the values of several important products are inclined downwards. In the latter category may be placed aspirin, salicylic acid, and salicylate of soda, the output of all of which drugs is steadily increasing. Quinine is now more like a normal market—at least as nearly normal as anything can be in war-time; the price is still materially above the level at which it stood before the recent boom, but holders are less firm in their views than they were last week. Salol continues its downward-price movement. Menthol is still in good demand at higher prices. Potassium permanganate is still scarcer and prices are advancing. The value of opium is inclined upwards, but makers of morphine have announced no change in their quotations. Apart from the synthetic drugs already mentioned, there is little change in the market position of any of them; phenacetin and barbitone continue to be in short supply.

